# State-of-the-art and future developments in dosimetry for SSTR- and PSMA-targeting radioligand therapies^☆^

**DOI:** 10.1016/j.zemedi.2026.03.011

**Published:** 2026-03-21

**Authors:** Sandra Resch, Astrid Delker

**Affiliations:** Department of Nuclear Medicine, LMU Hospital, Munich, Germany

**Keywords:** Dosimetry, Radioligand therapy, PSMA, PRRT

## Abstract

Radioligand therapy targeting the prostate-specific membrane antigen (PSMA) or somatostatin receptors (sstr) has become an established treatment option for metastatic castration-resistant prostate cancer and advanced or inoperable neuroendocrine tumours, respectively. Although several studies have already demonstrated the clinical usefulness of dosimetry-guided personalized treatment schedules, and although there is growing evidence for absorbed-dose-effect-relationships, the clinical implementation of personalized dosimetry is slow due to various factors, such as logistical constraints, a lack of standardisation or missing reimbursement. This review highlights current and future developments in quantitative imaging and personalized dosimetry for radioligand therapy. Current challenges, such as improving the accuracy and efficiency of quantitative imaging, imaging of alpha emitters, single-time-point dosimetry and PET-based absorbed dose prediction, microdosimetry and radiobiology, will be considered. Further, this review will address current ideas on how to implement dosimetry-guided treatment planning for radioligand therapy.

## 1 Introduction

With the FDA and EMA approval of Lutathera® and Pluvicto®, radioligand therapy (RLT) finally evolved as an established treatment option for advanced cancer disease, i.e. metastatic castration-resistant prostate cancer (mCRPC) and somatostatin receptor (SSTR)-positive gastroenteropancreatic neuroendocrine tumours (GEP-NETs), including foregut, midgut, and hindgut neuroendocrine tumours (NETs) [1]. Ongoing clinical trials on the investigation of alternative therapeutic radionuclides and new molecular targets will likely boost the number of clinically available treatment options in the near to mid future [2], [3], [4], [5]. Despite the progress in the field of RLT and theranostics, routine patient-specific dosimetry is still rarely established due to various factors, such as complicated logistics and missing reimbursement, missing uncertainty reporting, slow methodological standardization and slow dissemination of knowledge. This complicates to establish clinical multi-centre trials, which prove that using dosimetry-guided, individualized dosing schedules leads to improved patient outcome [6]. While within the current activity regimes acute toxicities may be to some extend inferred from clinical monitoring along with consecutive RLT cycles, dosimetry especially plays a major role in the management of long-term toxicities, that can arise over a time span of months to years after RLT and which become of increasing relevance as the life span of treated patients increases [7], [8]. In particular, the commonly applied absorbed dose thresholds for normal tissue toxicity are usually related to the probability of toxicities occurring within a certain timeframe after exposure to ionising radiation, such as a 5% probability within five years (TD5/5) [9]. Generating clinical evidence for dosimetry-guided treatment planning in RLT also includes to establish organ tolerances and tumour control probability curves specific for RLT.

An increasing number of studies already report absorbed-dose-effect-relationships (ADERs) for PSMA-targeting RLT and Peptid-Receptor-Radionuclide-Therapy (PRRT) targeting sstr. Ilan et al. established ADERs for pancreatic neuroendocrine tumours (NETs) based on 24 patients treated with [^177^Lu]Lu-DOTATATE [10]. Lesions were stratified according to size, accounting for size-dependent volumetric and quantitative uncertainties. Warfvinge et al. reported an ADER for CT-based tumour response in G2 NETs (69 tumours in 32 patients) after treatment with [^177^Lu]Lu-DOTATATE, for which the inclusion criterion for lesions was a minimum size of 4 ml [11]. Using 35 dosimetric datasets for consecutive cycles of [^177^Lu]Lu-DOTATATE treatment, Hebert et al. also reported ADERs for CT-based volume reduction in 146 lesions, as well as for absorbed doses to the bone marrow and spleen, and haematological toxicity [12]. Progression-free and overall survival were significantly prolonged in patients receiving a minimum cumulative lesion absorbed dose of approximately 52 Gy. Furthermore, they reported that the absorbed dose to most organs remained consistent between cycles, while a significant decrease in lesion absorbed dose for consecutive cycles was observed. Similar trends for longitudinal lesion dosimetry have been reported in other studies investigating PSMA- or sstr-targeting ligands [13], [14]. This highlights the potential for optimising treatment by adjusting the activity from cycle to cycle according to the expected lesion-to-organ absorbed doses. Regarding haematological toxicity after ^177^Lu-PRRT, several studies have reported moderate ADERs using image-based dosimetry [15], [16], [17]. Several studies have confirmed significant differences in lesion absorbed doses between biochemical or image-based responders and non-responders for PSMA-targeting radioligand therapy. However, exact cut-off values may vary according to differences in imaging and dosimetry methodology and reporting, as well as the patient cohort under investigation [13], [18], [19], [20]. Grkovski et al. reported significantly longer biochemical progression-free and overall survival for patients who received a higher mean lesion absorbed dose after [^177^Lu]Lu-PSMA-617 therapy [18]. They also found a moderate ADER for biochemical response and change in SUV_max_ in follow-up [^68^Ga]Ga-PSMA-11 PET/CT scans. Active bone marrow is also a major risk organ during mCRPC treatment, due to its high sensitivity to radiation and impaired haematological function in many patients, the latter which results from numerous pre-treatments and infiltration of metastases into the bone marrow. Furthermore, bone marrow metastases can cause significant cross-irradiation of the bone marrow. Rumiantcev et al. developed a methodology for image-based bone marrow dosimetry after [^177^Lu]Lu-PSMA therapy of mCRPC, finding significant and moderate ADERs for the course of erythrocytes, lymphocytes and thrombocytes [21].

The above studies emphasize the clinical potential of incorporating patient-specific dosimetry into RLT treatment planning. This review provides an overview of ongoing and near-future developments, which aim to at overcome the clinical challenges and limitations of routine patient-specific dosimetry and to provide reliable dosimetry to all patients. Furthermore, it addresses open questions regarding the ongoing development of RLT-specific ADER and current proposals for establishing dosimetry-guided RLT. The next two sections will provide an overview of radionuclides and ligands that are currently under investigation or in use, as well as an overview of the dosimetry methodology. Subsequent sections will address more specific research topics.

## 2 Ligands and radionuclides

The choice of radionuclide and targeting ligand fundamentally determines the dosimetric properties of RLT. Different radionuclides vary in their radiation type, energy, and range, which directly affects the absorbed dose at the organ, lesion, and cellular level. Similarly, ligand characteristics govern tumour and organ uptake, retention, and clearance, influencing both therapeutic efficacy and normal tissue exposure. Understanding the evolving landscape of radionuclides and ligands is therefore essential not only for expanding therapeutic options but also for improving the reliability of dosimetry-guided RLT.

Current developments on ligands and radionuclides are focusing on expanding the range of radiopharmaceuticals beyond the well-established ones, i.e. [^177^Lu]Lu-PSMA and those used in ^177^Lu-PRRT. Table 1 provides an overview of the radionuclides, ligands and production routes currently in use or under further investigation. There is a particular focus on targeted alpha therapy (TAT) using radionuclides such as ^225^Ac, ^212^Pb, ^211^At and ^149^Tb (Table 1). Alpha particles are characterized by a high linear energy transfer in the range of approximately 50-230 keV/µm and a range of 50-100 µm in soft tissue, which leads to a high probability of causing complex, irreparable DNA damage [22]. Recent studies on PSMA-targeted RLT include [^211^At]At-PSMA-5 and [^212^Pb]Pb-ADVC001, both of which are moving towards the first in-human therapies [23], [24]. Many α-emitters, however, have challenging decay characteristics, present difficulties in radiation protection or are limited in supply. For example, ^225^Ac exhibits multiple α-emissions with recoil energies that can cause dissociations of the daughter nuclides from the chelator [25]. Furthermore, global production and availability of ^225^Ac is still limited, although new production routes and industrial partnerships are in development [26], [27]. ^212^Pb and ^211^At face radioprotection challenges: during the decay of ^212^Pb, a 2.6 MeV gamma photon is released and ^211^At is a halogen with high volatility similar to ^131^I [28], [29].

**Table 1:** Overview of current radionuclides and ligands in human use

Radio-nuclide	Main decay type	Half-life	Mean soft tissue range	Main ligands	Production	Comments
^177^Lu	β^-^	6.7 d	0.67 mm [40]	DOTATATE (Lutathera®), DOTATOC, DOTA-LM3 [41], PSMA-617 (Pluvicto®), PSMA-I&T, EB-PSMA [42]	Nuclear reactor	Widely used
^90^Y	β^-^	2.7 d	2.5 mm [43]	DOTATATE, DOTATOC, PSMA-617	Nuclear reactor	High energy β^-^-emission
^188^Re	β^-^	16.9 h	3.3 mm*	PSMA-GCK01	Generator	Theranostic pair with ^99m^Tc
^161^Tb	β^-^	6.9 d	0.4 mm**	DOTATATE, DOTATOC, DOTA-LM3, PSMA-617 [44], PSMA-I&T [45]	Nuclear reactor	Similar to ^177^Lu, but additional Auger and conversion electrons
^225^Ac	α	9.9 d	47 – 85 µm [46]	DOTATATE [47], DOTATOC [48], PSMA-617 [49], PSMA-I&T [50]	Generator, Accelerator	Recoil from daughters has to be considered
^212^Pb	α	10.6 h	40 –100 µm [22]	DOTAMTATE [51], ADVC001 [24] and AB001 [52] (both PSMA-targeted)	Generator	Theranostic pair with ^203^Pb
^211^At	α	7.2 h	55 – 80 µm [22]	MX35 F(ab')_2_[53], PSMA-5 [23]	Cyclotron	^207^Bi in decay chain (38 years half-life)
^149^Tb	α	4.1 h	∼ 25 µm [54]	-	Spallation	Only preclinical at the moment

* NIST CSDA range with mean electron energy of ^188^Re decay at 784 keV [37], [38].

^**^NIST CSDA range with mean electron energy of ^161^Tb decay at 202.5 keV [38], [39].

While ^177^Lu has become the standard β^-^-emitter for both SSTR- and PSMA-targeted therapies, ^90^Y has been studied for treatment of NETs (e.g. [^90^Y]Y-DOTATOC). ^90^Y emits a higher β^-^-energy than ^177^Lu (maximum energy of approximately 2.3 vs. 0.5 MeV), which makes it more beneficial for treatment of larger tumours. However, ^90^Y is also associated with a higher risk of renal toxicity and quantitative imaging is more challenging [7], [30].

Another β^-^-emitter of renewed interest is ^188^Re, a generator-produced radionuclide with a high maximum β^-^-energy of 2.1 MeV and a half-life of 16.9 h [31]. Its availability from an ^188^W/^188^Re generator makes it an appealing candidate for widespread therapeutic use, particularly in settings with limited access to reactor-produced radionuclides. A recent study by Cardinale et al. has evaluated the ^99m^Tc/^188^Re-PSMA-GCK01 theranostic pair pre-clinically but also in first in-human cases, which has confirmed its feasibility [31].

Meanwhile, ^161^Tb is being investigated more and more as a medium-energy β^-^-emitter with additional Auger and conversion electron emissions. These low-range electrons have LET values of approximately 4-26 keV/µm, which are substantially higher than the 0.1-1.0 keV /µm typical for β^-^-. The maximum β^-^-energies, however, are quite similar with 590 keV for ^161^Tb and 497 keV for ^177^Lu [32], [33]. Pre-clinical and simulation studies have demonstrated the advantages of ^161^Tb over ^177^Lu. For example, Alcocer-Ávila et al. demonstrated in a simulation study that the absorbed dose in single cells and micrometastases is higher for ^161^Tb than for ^177^Lu [34]. In the Violet trial (a single-centre, single-arm phase I/II study), patients with mCRPC who had progressed after taxane chemotherapy and androgen-receptor–targeted therapy were treated with [^161^Tb]Tb-PSMA-I&T [35]. Interim results showed that 70% of patients achieved a PSA reduction of more than 50 %.

Further, ^161^Tb can be used within the so-called Terbium quadruplet (^149^Tb, ^152^Tb, ^155^Tb and ^161^Tb), which is an isotope set ideal for theranostic applications [36]. Diagnostics and pre-therapeutic dosimetry can be achieved using either ^155^Tb for Gamma camera imaging or ^152^Tb using Positron-Emission-Tomography (PET), while ^149^Tb is a promising candidate for targeted alpha therapy.

## 3 Patient-specific dosimetry

This section will provide an overview of the key steps in the dosimetry chain. Subsequent sections will then describe current and future efforts relating to these steps in more detail.

Patient-specific dosimetry requires quantitative nuclear medicine imaging in order to measure the activity distribution within patients in units of Becquerel (Bq) (MIRD 23) [55]. Most of the therapeutic radionuclides currently in use emit gamma photons that can be imaged using a Gamma camera via 3D SPECT (single-photon-emission-computed-tomography) or 2D planar scintigraphy. Due to reduced tissue overlap and the ability to correct for image-degrading effects (e.g. photon absorption and scatter) during the reconstruction process, SPECT-based dosimetry protocols (SPECT-only or hybrid SPECT-planar) are becoming increasingly favoured [14], [56]. However, even when SPECT-based dosimetry protocols are used, quantitative imaging remains a major contributor to dosimetric uncertainty, particularly for small structures (e.g. small lesions, salivary glands) [57].

The total number of decays occurring in a given tissue is directly proportional to the amount of absorbed dose (unit: Gray (Gy)) deposited by the decay particles. The absorbed dose is given by:

(1) D=E/m;[D]=[J/kg]=[Gy]

with E being the energy deposited by the decay particles and m the mass of the tissue of interest. E is dependent on both, the physical characteristics of the radionuclide (decay type, energy spectrum, particle range, etc.) and the geometry (e.g. tissue diameter in comparison to the characteristic particle range), while m is dependent on the geometry and the tissue composition [58], [59]. As informed from radiobiological experiments, the physical radiation-absorbed dose shows a correlation with cellular survival after radiation exposure. Extensions of this concept exist to improve this correlation, e.g. considering the particle type (RBE-weighted dose) or the rate and fractionation at which the absorbed dose is delivered to the tissue (biologically effective dose (BED)) [60].

To determine the total number of decays that occur in a specific tissue-of-interest over time, the patient-specific pharmacokinetics of that tissue must be measured. As PSMA-targeting RLT or PRRT is usually applied systemically, a series of quantitative images must be acquired at the appropriate time points after injection [61]. The number of acquired time points affects both, the required clinical workload and the accuracy of absorbed dose estimates, and therefore these two aspects must be constantly balanced against each other. As will be described below, much recent research has focused on estimating the patient-specific pharmacokinetics more efficiently. Examples include combining single image acquisitions with prior pharmacokinetic knowledge, using novel imaging hardware, or optimising image acquisition, reconstruction and post-processing protocols.

Once the time-activity data is acquired, the complete time-activity-curve (TAC) from injection on up to the last decay has to be modelled by combining the sampled time-activity data with an appropriate mathematical curve model [61]. The mathematical curve model must be selected appropriately for the sampling scheme and vice versa. Although radiopharmaceuticals typically exhibit complex pharmacokinetics, not all phases may be equally important for determining the absorbed dose with sufficient accuracy. In kidney dosimetry after ^177^Lu-PRRT, for example, the fast pharmacokinetic phase observed immediately after injection typically contributes only a few percent to the total absorbed dose. However, if a data point is sampled in that early phase and mixed with data points from a later phase, the TAC describing the late, slow kidney pharmacokinetics can be falsified [62].

Given the tissue TAC, integrating this TAC from zero (injection) to infinity (last decay) provides the so-called time-integrated activity (TIA), which represents the total number of decays that occurred in the tissue under consideration. The TIA can be converted to absorbed dose, for example, by applying conversion factors (S values) tabulated for various radionuclides and geometric models (humans or animals) [63], [64]. S values are publicly available, e.g., via the OpenDose project [64]. S values can provide a reasonable estimate for the mean absorbed dose if the following criteria are met: 1. for radionuclides emitting mainly short-range particles (e.g. ^177^Lu or ^225^Ac); 2. for tissues with a strong self-accumulation of the radiopharmaceutical (self-absorbed dose is the dominant contributor; e.g. lesions, kidneys); 3. appropriate density scaling is applied; 4. the characteristic length scales are larger than the particle range [65]. 3D, i.e. voxel-level, dosimetry can be achieved using absorbed dose kernels, voxel S values or Monte Carlo simulations. These methods can account for the patient-specific 3D activity distribution, while Monte Carlo simulations fully account for the patient-specific 3D tissue characteristics in addition. Voxelised absorbed dose kernels can be found, e.g. via [66]. Monte-Carlo-based absorbed dose calculations are implemented in several dedicated simulation frameworks, as for example GATE (Geant4 Application for Tomographic Emission) or FLUKA [67], [68]. For full 3D dosimetry, the mathematical curve model can be fitted either to the voxel-wise time-activity data or to the 3D absorbed dose rate maps. It is still an open research field, to which extend 3D dosimetry can contribute towards personalised RLT, considering also that 3D absorbed dose distributions can be degraded by the limited spatial resolution of SPECT imaging, image noise and artefacts, as well as errors in voxel-wise co-registration [69]. However, 3D dosimetry has been found to be advantageous for bone marrow dosimetry in [^177^Lu]Lu-PSMA RLT for mCRPC and ^177^Lu-PRRT, e.g., where cross-absorbed doses are a dominant contributor to the total absorbed dose and tissue heterogeneity is high [15], [70]. Further, the distribution of bone lesions – if present - is also highly patient-specific and cannot be accounted for via tabulated S-values.

## 4 Quantitative imaging

Quantitative gamma-camera imaging, and in particular quantitative SPECT/CT forms the technical foundation of patient-specific dosimetry in PSMA-targeting RLT and PRRT. The following section summarizes the essential components of quantitative SPECT acquisition and reconstruction and highlights current developments that are particularly relevant for β^-^- and α-emitting radiopharmaceuticals. More precisely, the focus is placed on reconstruction methods, camera calibration, corrections for image-degrading effects, strategies for accelerating ^177^Lu SPECT acquisitions, and recent advances enabling quantitative imaging of α-emitters.

### 4.1 Reconstruction

Image reconstruction is an essential part towards quantitative SPECT and transforms measured two-dimensional projection data into three-dimensional quantitative images in units of Bq or Bq/ml. The current state-of-the-art is iterative reconstruction, with the ordered-subset-expectation-maximization (OSEM) algorithm being the most commonly used. OSEM accelerates the classical Maximum-Likelihood-Expectation-Maximisation (MLEM) algorithm by dividing the projection data into subsets, which speeds up the reconstruction speed. However, OSEM tends to amplify noise in the reconstructions due to noise being present in the measured projections. Thus, the number of iterations is often limited such that the algorithm is stopped before full convergence can be reached. For further noise reduction or to also suppress image artefacts (e.g. Gibb’s ringing artefacts), image regularization can be applied, e.g. in form of post-filtering or dedicated maximum a priori (MAP) reconstruction algorithms [71]. Within MAP reconstruction, different penalty functions can be used, e.g., the smoothing quadratic penalty or the more edge preserving relative difference penalty [72], [73]. Further priors include anatomical data or PET images, which have been shown to improve RCs and reduce overshoots when incorporated into hybrid kernelized expectation maximisation algorithm [74]. An alternative to purely analytical iterative reconstruction is to use Monte Carlo–based reconstruction, in which a full physical model of photon transport (i.e. scattering and attenuation) and detector response is used in the forward projection step. In a recent meeting report, Polson et al. presented a fully Monte-Carlo-based SPECT reconstruction framework implemented in PyTomography (using SIMIND as Monte Carlo backend), highlighting renewed efforts to make Monte-Carlo-based reconstruction practicable even for clinical SPECT imaging [75]. Earlier work by Rydén et al. demonstrated that Monte-Carlo based ^177^Lu-SPECT reconstructions can be accelerated by GPU parallelization to 3 minutes [76].

### 4.2 Camera calibration

A crucial step in the absorbed dose calculation process is the activity quantification itself since errors in this step will propagate into dosimetry. In quantitative SPECT, a calibration factor (CF) is used to convert detected counts per second and per voxel into units of activity or activity concentration:

The standard approach for calculating the CF is to perform a phantom measurement with a known activity concentration, measured in an independently calibrated activity meter [77]. Usually, a SPECT/CT of a large, uniform phantom, often of cylindrical shape, is acquired and reconstructed using identical parameters as used in patient scans. After reconstruction, a VOI within the phantom is drawn from which the count rate per volume is extracted and compared to the known activity concentration:

(2) $$\mathrm{CF}\;=\;count\;rate\;in\;VOI\;\left[\mathrm{cps}/ml\right]/known\;activity\;concentration\;in\;VOI\;\left[\mathrm{MBq}/ml\right]$$

Since the CF usually reflects not only the camera’s detector characteristics (including collimator), but also specific conditions within the acquisition protocol (e.g. extreme low count measurements) and the reconstruction, it is inherently camera- and protocol-specific**.**

To harmonize quantitative ^177^Lu-SPECT imaging across multiples sites, an EARL accreditation study has been under development since March 2022 [78]. Substantial efforts towards standardization of ^177^Lu-SPECT/CT imaging have been made within the MRTDosimetry project, a combined European initiative of metrology and nuclear medicine research. In a multi-centre evaluation, Tran-Gia et al. compared CFs, partial volume correction (PVC) and the validation of the quantitative imaging setup from nine SPECT/CTs [79]. They demonstrated that, under harmonised acquisition and reconstruction conditions, CFs were consistent for systems with the same camera model and reconstruction software indicating that comparable measurement sensitivities can indeed be achieved between sites. Their work also highlighted that partial-volume effects (PVE) remain one of the dominant sources of uncertainty: even after applying volume-based recovery coefficients (RCs), systematic under- or over-estimation persisted for small or complex organ volumes. This underscores that, while cross-site harmonisation of calibration is feasible, accurate dosimetry still requires careful validation of PVC strategies, which will be elaborated in the following Section 4.3.

### 4.3 Corrections for image-degrading effects

The accuracy of quantitative imaging depends on various factors, such as radionuclide, object volume and shape, object-to-background ratio, scanner calibration or the reconstruction settings used. The ongoing improvement of corrections for image-degrading effects - either as part of the reconstruction itself or as a post-processing - is another crucial component towards improving patient-specific dosimetry.

The finite spatial resolution remains one main fundamental limitation in quantitative SPECT imaging and thus, patient-specific dosimetry. More precisely, the limited spatial resolution leads to activity spill-out and spill-in (spill-over), with the consequence of erroneous activity measurements in small objects (e.g. lesions, salivary glands). This is often referred to as one source of PVE [80]. In SPECT imaging, the spatial resolution, i.e. the system’s point-spread-function (PSF), is mainly driven by the radionuclide and collimator, both of which determine the distance-dependent collimator-detector-response (CDR) [55]. Although many commercially available reconstruction algorithms nowadays allow modelling of the distance-dependent CDR (also referred to as resolution compensation or point spread function (PSF) modelling), the spatial resolution remains limited. This is partly because signal information is lost during the measurement process, and partly because of imperfect corrections. Due to its importance for reducing uncertainties in patient-specific dosimetry, PVC is still an active research topic and has been investigated in many recent studies.

The classical approach to PVC, which is still widely applied due to its simple implementation, relies on phantom-based correction factors. Therefore, RCs are derived from phantom measurements including objects with known size, shape and activity resulting in a volume-to-RC calibration curve. Classically, this was done using the NEMA IEC body phantom with spherical inserts of various volumes. To then correct for an object’s PVE, the volume of the object is estimated, and the measured SPECT activity is multiplied with a correction factor as derived from the predefined volume-to-RC calibration curve. However, several recent studies have demonstrated that RCs depend not only on object volume but also on shape, surface area and background activity. Mínguez Gabiña et al. have shown theoretically and with phantom measurements of spheres, spheroids and oblate spheroids that the signal rate per activity varies with object shape, but when characterized by the volume-to-surface ratio (rather than simply volume-equivalent radius) the signal behaviour across different shapes follows a consistent trend [81]. Grings et al. reported similar findings investigating 19 three-dimensional printed kidney phantoms, demonstrating that the surface-area-to-volume-ratio correlates highly with RCs [82]. They highlighted again that PVC in patient SPECT images with simple spherical shapes may be insufficient. This has motivated the development of adapted corrections. For example, Marquis et al. proposed two shape-specific RC models under the MIRD Committee framework, which showed major quantification improvements for ellipsoid shapes for which the quantification error was reduced by factors from 1.7 to 4.0 for ^177^Lu compared to classical RC curve corrections [83].

Beyond volume- or shape-based RC corrections, alternative PVC strategies aim to correct for PVE directly using the system’s point-spread-function or object-specific segmentations. Liu et al. investigated the reblurred Van-Cittert as iterative deconvolution technique and iterative Yang for PVC in [^177^Lu]Lu-PSMA SPECT imaging [84]. Iterative Yang performed best for small tumours but requires precise object segmentations and anatomical alignment, while reblurred Van-Cittert was more sensitive to noise and ringing artifacts. Leube et al. introduced deep-learning-based PVC, which was shown to be superior to PVC using the iterative Yang method [85]. Rumiantcev et al. investigated PVC to reduce the activity overestimation close to bone lesions for bone marrow dosimetry after [^177^Lu]Lu-PSMA therapy of mCRPC, with the best performance being observed for iterative Yang with SPECT-based lesion segmentation [21]. However, full PVC was not possible and authors recommend to base bone marrow dosimetry on lesion-free skeletal sites, if possible. Peters et al. applied an oversize-VOI approach to consider the activity spill-over of small lesions in patients with hormone-sensitive prostate cancer, who received treatment with [^177^Lu]Lu-PSMA-617 [86].

Apart from PVE, inaccurate scatter correction can significantly impair quantitative accuracy [87]. Triple-energy-window scatter correction is one of the most commonly applied methods for ^177^Lu-SPECT since it is simple to implement and integrated in many commercial reconstruction software. It uses energy windows placed adjacent to the photopeak to estimate scatter within the photopeak [55]. This correction typically performs well in tissues with high uptake, such as kidneys, but its accuracy decreases in regions with low activity concentrations, e.g. in bone marrow regions or for late measurements. Robinson et al. demonstrated that for activities ≤ 200 MBq, TEW correction leads to a substantial overestimation of the total recovered activity [87]. Monte-Carlo-based scatter estimation provides a more physically accurate modelling of scatter interactions, at the cost of increased computational burden. To overcome this, accelerated Monte Carlo techniques, such as those proposed by Hippeläinen et al., have been introduced, and fully Monte-Carlo-based GPU-accelerated reconstruction frameworks, e.g. developed by Rydén et al., have shown promising performance [76], [88].

Further, the accuracy in SPECT can be significantly compromised by the image blurring and artifacts introduced through patient motion, which includes irregular motion (e.g. pain-driven patient shifts) and regular motion (e.g. periodic cardiac or respiratory motion) [89], [90]. While motion correction is more commonly implemented in PET, its implementation in SPECT imaging is still an open research field. Since external motion tracking devices may be expensive and complex to use, estimation of motion directly from the acquired raw data using data-driven techniques is preferable. One such example is the tomographic consistency correction (MC Pro) by Siemens, as elaborated by Reymann et al. in a recent white paper [91]. Motion effects should be minimized already during the SPECT acquisition by optimizing patient positioning (e.g. vacuum pillows, clothe belts), while remaining motion-related misalignment between SPECT projections should be corrected retrospectively. Further, similar patient positioning from one SPECT acquisition to another facilitates co-registration of individual time points and thus automatic dosimetry workflows.

Dead time corrections are less frequently employed, especially for ^177^Lu SPECT imaging. However, Desy et al. showed in two studies that dead time has an impact on quantitative ^177^ Lu-SPECT especially for personalized therapies with higher administered activities (e.g. up to 33.7 GBq in that study) [92]. They propose a practical post-reconstruction per-volume correction in a later publication [93].

### 4.4 Fast scanning for ^177^Lutetium

Quantitative SPECT imaging for therapies involving ^177^Lu-labeled radioligands is well established, however, routine integration of dosimetry is often limited by the high workload and device usage associated with multiple SPECT or SPECT/CT acquisitions. While recent studies have explored reducing the number of imaging time points (cf. section on single-time-point dosimetry), an alternative strategy would be to shorten the duration of individual SPECT acquisitions. Whole-body SPECT acquisitions would be favourable to cover all relevant risk organs as well as the entire tumour mass for dosimetry purposes. At the same time, whole-body SPECT acquisitions—typically involving three to four detector positions—still require more than one hour total acquisition time in many clinical settings [94], [95], [96], [97]. Thus, recent efforts focus on accelerating scan protocols without compromising image quality or dosimetric accuracy.

A direct approach would be to reduce the acquisition time per projection. Ivashchenko et al. showed for ^177^Lu-PSMA therapy that projection times could be reduced from 14 s to 3.5 s on day one post treatment, with minor deviations in kidney quantification (3.5 ± 4.4 %) [98]. Despite lower count rates on day seven post injection, an acquisition time of 10.5 s per projection still achieved acceptable accuracy (3.3 ± 6.7 %). To support a reduction of acquisition times, image denoising approaches can be applied. One promising example is a deep-learning-based method developed for [^177^Lu]Lu-PSMA three-bed SPECT imaging, trained using [^68^Ga]Ga-PSMA-PET/CT-derived virtual SPECT data [99]. The denoised patient reconstructions performed well in a visual evaluation and the phantom reconstructions showed reduced coefficients-of-variation, but also a worse signal-to-background ratio. Furthermore, Zaragori et al. investigated a reduction of the acquisition time from 32 minutes for two bed positions on a conventional SPECT/CT to 18 minutes for a whole-body scan on a CZT SPECT/CT (Veriton, Spectrum Dynamics Medical, Sarasota, Florida, US) [100]. The median absorbed doses of the largest tumour lesions in 12 patients treated with ^177^Lu-DOTATATE did not show significant differences, with 1.8 (0.9 – 5.7) Gy/GBq for the conventional Symbia T2 SPECT/CT system (Siemens Healthineers, Erlangen, Germany) vs. 2.0 (1.0 - 7.3) Gy/GBq on the CZT SPECT/CT. The potential of using CZT SPECT systems for quantitative SPECT imaging in RLT was also demonstrated in another study by Nuttens et al. [101].

Another approach to shorten the total acquisition time would be to reduce the number of acquired projections. Rydén et al. explored whether deep learning could generate synthetic intermediate projections for ^177^Lu-SPECT [94]. By reducing the number of measured projections from 120 to 30 and using a convolutional neural network to synthesize missing intermediate views, they achieved reconstructed images with kidney activity differing by only 3% compared to the full-view datasets.

Thus, the mentioned studies demonstrate that current acquisition times for quantitative SPECT for PSMA-targeting RLT or PRRT using ^177^Lu can be substantially optimised, by reducing acquisition times per projection particularly in early post-treatment scans or by predicting intermediate projection data. Image denoising plays a major role for lower count rates, in particular for SPECT acquisitions at late time points post injection.

### 4.5 Quantitative imaging of alpha-emitters: From feasibility to first in-human studies

Quantitative SPECT imaging of α-emitters remains challenging because therapeutic activities are low and many relevant radionuclides exhibit complex decay chains with high-energy photons that cause septal penetration and strong scatter contribution in lower energy windows [102]. Despite these limitations, recent work has demonstrated that quantitative imaging is feasible with adapted acquisition protocols and reconstruction methods.

Clinical imaging of ^225^Ac-labelled tracers was first reported in 2020 for [^225^Ac] ^225^Ac-DOTATATE, followed by the first quantitative SPECT/CT images of [^225^Ac] ^225^Ac-PSMA-I&T in 2021 [103], [104], [105], [106]. These studies showed that imaging at the 440 keV photopeak of the ^213^Bi daughter is possible when using high-energy collimators and long acquisition times (∼210 s per projection/∼1 h in total) [106]. A subsequent study by Liubchenko et al. included serial SPECT/CT imaging at 24 h and 48 h p.i. which used both, the 440 keV (^213^Bi), 218 keV (^221^Fr) as well as an energy window centered at 78 keV to capture the X-ray emissions [107]. Reconstruction included a MAP-EM algorithm with transmission-dependent scatter correction and resolution modelling via a simulated 2D distance-dependent PSF.

Beyond clinical pilot data, phantom studies have further explored the performance limits of ^225^Ac SPECT. Tulik et al. demonstrated that quantitative accuracy within roughly 10 % is achievable, although background signal from septal penetration and insufficient scatter correction was observed [108]. The study highlights the feasibility of imaging with ^225^Ac, however, authors claim that optimal quantitative imaging requires reconstruction algorithms with, e.g., specialized scatter correction, which may not yet be commercially available.

After a first proof of concept study by Kvassheim et al. on the feasibility of quantitative SPECT imaging of ^212^Pb in 2022 using different imaging protocols, the first SPECT images of a patient treated with [^212^Pb]Pb-PSMA were shown in 2024 by Griffiths et al. [109], [110]. For imaging, the energy windows at 239 keV (width 10 %) and 79 keV (width 20 %), high-energy collimators and 30 s acquisition time per projection were used. In a recent study by Kästner et al., gamma camera imaging of the theranostic pair ^203^Pb/^212^Pb has been systematically investigated and compared in a phantom study, demonstrating clinical feasibility for both isotopes, but also the current challenges associated with direct ^212^Pb gamma camera imaging. These include scatter, septal penetration and dead time count losses especially in the 79 keV energy window [111].

Similar progress has been made for ^211^At: Already in 2015, Cederkrantz et al. showed the feasibility of SPECT imaging of patients with ovarian cancer treated with [^211^At] ^211^At-MX35 F(ab')2 based on X-ray emissions following electron capture [112]. Imaging was performed with a medium energy collimator, an energy window centered at 79 keV (width 15 %) and 20-30 s acquisition time per projection. Effective-scatter-source-estimation and collimator response compensation, both established using Monte Carlo simulations, were included in the reconstruction [113].

Overall, these early clinical and phantom studies indicate that quantitative SPECT imaging of α-emitters is feasible but requires tailored acquisition settings and reconstruction methods incorporating advanced scatter and detector response modelling.

## 5 Single-time-point dosimetry

As discussed, dosimetry in RLT ideally requires multiple imaging sessions over time, with two to three acquisitions per considered pharmacokinetic phase to model the TAC in tumors or organs [96]. However, this is often challenging to realise in clinical settings due to multiple factors, such as logistical constraints, high patient burden especially in case of out-patient examinations, limited scanner availability or lack of reimbursement. These limitations have led to a growing interest in simplified dosimetry approaches, which aim to reduce the number of imaging acquisitions significantly, potentially down to a single post-therapeutic acquisition (single-time-point (STP) dosimetry), while maintaining acceptable dosimetric accuracy. In recent years, numerous studies have investigated STP dosimetry, especially for ^177^Lu-based therapies, mainly relying on population-based knowledge about lesion or organ effective half-lives, or on patient-specific lesion or organ effective half-lives acquired in a previous treatment cycle [114], [115], [116]. The patient-specific lesion or organ uptake of the respective therapy cycle is then assessed via a single reference image acquisition. A limitation of these methods is that they do not account for inter-patient or inter-cycle pharmacokinetic variability, which can be for example due to differences in organ function, tumor burden or prior treatments.

To maintain appropriate patient specificity, recent work focused on more individualized STP dosimetry, such as non-linear mixed-effects (NLME) or physioligically-based pharmacokinetic modelling, incorporating patient-specific biomarkers or usage of pre-therapeutic PET data. Both, Devasia et al. and Hardiansyah et al., showed that NLME model fitting in STP dosimetry of [^177^Lu]Lu-DOTATATE and [^177^Lu]Lu-PSMA therapy provides a more accurate estimation of the renal TIA than solely population-based methods [117], [118]. Hardiansyah et al. additionally performed population-based model selection. Chicheportiche et al. investigated wether absorbed doses during [^177^Lu]Lu-DOTATATE treatment can be estimated from a single SPECT/CT using a multiple linear regression model [119]. The model was trained with 40 patients with NET and subsequently tested in 32 additional patients. In this test cohort, the differences in cumulative absorbed dose compared with the standard multi-time-point method were generally small, with e.g. 3.5 % ± 19.3 for tumors using a single SPECT/CT at 168 h p.i. in the first therapy cycle and at 24 h p.i. in all following cycles.

However, as noted by Sadeghi et al., these advanced approaches can be challenging because they are computationally intensive, complex to implement, and require large datasets for model selection and validation [120]. Further, they propose a flexible STP dosimetry approach that adapts the imaging time point to individual patient characteristics and radioligand types. Other studies have also investigated methods, that allow for more flexible selection of imaging time points as discussed in a study by Wang et al., for example [121]. The authors developed a patient-specific regression model to predict the TIA from a single SPECT/CT time point, also incorporating input features such as the estimated glomerular filtration rate (eGFR) or total tumor burden. This data-driven STP-dosimetry approach resulted in a higher flexibility in the selection of the imaging time point. Their approach provided dosimetry estimations with a mean absolute error of less than 12 % for kidneys and 27 % for tumors based on SPECT/CT imaging at day one post injection [121].

In any case, the imaging time point always represents a compromise between optimising dosimetry for lesions and organs-at-risk [122]. Many studies so far focused on optimising the imaging time point and the STP dosimetry method for kidney dosimetry [118], [119], [121]. In the future, recommendations for the feasibility of STP dosimetry per patient may be based on patient-specific clinical parameters, for example, low eGFR values or a high total tumour burden, that may lead to deviations in pharmacokinetics compared to the population mean and thus could be contraindications for STP dosimetry.

## 6 PET-based absorbed dose prediction

While dosimetry-guided treatment planning in EBRT is based on pretherapeutic dosimetry, with adjustments made to the individual plan between fractions, if necessary, personalised dosimetry in RLT is so far primarily a post-therapeutic procedure. Therapeutic activities employed in successive treatment cycles could then be adjusted according to the patient-specific pharmacokinetics and dosimetry of, e.g, the first therapy cycle. However, using higher administered activities already in the first treatment cycle may be of special interest, for example to exploit higher lesion-to-organ absorbed dose ratios [12], [13], [14]. As patients usually undergo PET imaging prior to RLT using the same molecular target, this could enable estimation of the pre-therapeutic absorbed dose by using the PET as a surrogate for the post-therapeutic patient-specific pharmacokinetics. The main limitation of this approach is that in routine praxis only a single static PET image is usually available, acquired within the first few hours after injection. This especially complicates the prediction of the absorbed doses to lesions, which usually show a more variable and slower pharmacokinetics compared to organs. Based on the analysis of ten patients with hormone-sensitive prostate cancer, Peters et al. reported better agreement for organ than lesion dosimetry based on [^68^Ga]Ga-PSMA-11 PET compared to dosimetry based on serial SPECT/CT data acquired 1, 24, 48, 72 and 168 h after administration of [177Lu]Lu-PSMA-617 [86]. Peterson et al. investigated five PET-related uptake metrics along with seven non-imaging biomarkers in a cohort of 25 patients, with the aim to predict renal absorbed doses after treatment with Lutathera®. They found a mean absolute percent deviation of 18 % for kidney dosimetry using an univariable regression model based on [^68^Ga]Ga-DOTATATE PET uptake ((Bq/ml)/MBq), showing also the best performance among all investigated parameters. A bivariable regression model including eGFR lead to a minor improvement of absorbed dose prediction (mean absolute percent deviation: 17.3 %) [123]. Xue et al. investigated random forest regression and a 4-layer neural network to predict organ absorbed doses after [^177^Lu]Lu-PSMA-I&T therapy using the pre-therapeutic [^68^Ga]Ga-PSMA-11 PET and achieved a mean absolute percent error of 15.8 ± 13.2 %, 29.6 ± 13.7 %, 23.8 ± 13.1 %, and 32.1 ± 31.4 % for kidneys, liver, salivary glands and spleen in a cohort of 23 patients [124]. In another study, Xue et al. additionally investigated the feasibility of predicting 3D absorbed dose distributions using a generative adversarial network, which resulted in a voxel-wise, normalized root-mean-squared-error of 1.7 ± 0.4 % and a structural similarity index measure of 0.94 ± 0.03 compared to the post-therapeutic 3D absorbed dose distribution [125].

To in particular capture long retention in lesions, longer-lived radionuclides, such as ^89^Zr with a half-life of 3.2 days or 64Cu with 12.7 hours, could be of advantage for pre-therapeutic dosimetry, while a trade-off between predictive accuracy and the number of pre-therapeutic PET scans may be necessary [126], [127], [128]. It has been shown by Privé et al. that the biodistribution data from multi-time-point ^89^Zr-PET-imaging (1, 3, 24, 48 h p. i.) could be used to predict [^177^Lu]Lu-PSMA pharmacokinetics in lesions [127].

The aforementioned studies highlight the promise and current limitations of PET-based pre-therapeutic absorbed dose prediction. Further studies on larger patient cohorts and more theranostic pairs are needed, and future methodological development may substantially benefit from advanced modelling approaches to improve clinical accuracy and applicability in personalised RLT dosimetry. In silico clinical trials could complement prospective multicentre trials in assessing the potential of PET-based predictive dosimetry for improving patient selection and adjusting dosing regimens with the start of the first treatment cycle.

## 7 Microdosimetry and radiobiology

Image-based clinical dosimetry using SPECT or PET is inherently limited to a spatial resolution of a few millimetres or centimetres. However, as RLT relies on pharmacological principles to enable a systemic and targeted radiation delivery to tumour cells, it is naturally associated with a highly heterogeneous activity distribution at the microscale [129], [130]. The microdistribution of the radiopharmaceutical, in combination with the tissue and cellular geometry, as well as the characteristic particle range of the radionuclide, determines the likelihood of induced DNA damage. Thus, the design of the radiopharmaceutical must be particularly appropriate for very short-ranged particles, such as Auger electrons or alpha particles, in order to bring them as close as possible to the cell nucleus and DNA as the critical target [131], [132]. Microdosimetry is a key element for a better understanding of the physical, radiochemical and radiobiological properties of RLT. For example, Peter et al. used 3D digital autoradiography acquired with an iQID camera (QScint Imaging Solutions, LLC, Tucson, Arizona, US) to perform 3D tumour and kidney small-scale dosimetry of [^225^Ac]Ac-Macropa-PEG4-YS5 in mice [133]. Further, reliable microdosimetry is a major aspect in determining radiobiological quantities. However, microdosimetry for preclinical in-vitro, in-vivo or ex-vivo studies is particularly challenging, as self-absorbed and cross-absorbed doses are strongly dependent on the specific geometry of single cells or cellular sub-compartments, the 2D or 3D cell or tissue geometry as well as the micro distribution of activity [134], [135]. Thus, future efforts must assess the associated uncertainties and their implications on the RLT-specific radiobiological knowledge.

Another challenge in current clinical dosimetry practice for PSMA-targeted RLT and PRRT is that - although dosimetry concepts considering the physical and radiobiological characteristics of the radiopharmaceutical are available (e.g. relative biological effectiveness (RBE) or BED)- the necessary radiobiological quantities (e.g. alpha/beta ratios) are currently mostly obtained from EBRT. However, EBRT is a radiation modality that differs in multiple ways from RLT, such as absorbed dose rate, fractionation or uniformity of the radiation field [136]. Thus, future efforts in the field of RLT should include preclinical in-vitro, in-vivo and ex-vivo studies – complemented by in-silico models - to obtain a comprehensive understanding of RLT-specific radiobiological effects. Tamborino et al. recently published a workflow for characterizing the response and radiobiological quantities (α, β values) for the GOT1 NET and the NCI-H69 small-cell lung cancer cell lines after [^177^Lu]Lu-DOTATATE treatment in-vitro, compared to EBRT [134]. The authors report that a three to four times higher absorbed dose is required for PRRT in comparison to obtain a comparable treatment response. Ruigrok et al. investigated the RBE of [^225^Ac]Ac-PSMA-I&T compared to [^177^Lu]Lu-PSMA-I&T based on in-vitro survival assays of the PC3-PIP cell line after 3 h of incubation time [137]. The RBE is intended to account for the higher cytotoxicity of, e.g., alpha emitters within the concept of RBE-weighted dose [59], [138]. Ruigrok et al. reported an RBE of 4.2, which is in the range recommended so far for targeted alpha therapy [137], [138]. However, the RBE depends on the biological endpoint being considered and the reference radiation. Thus, in future, RBE factors determined in preclinical in-vitro or in-vivo conditions should be related to those based on clinical endpoints such as overall survival or progression-free survival. In-silico studies, such as those carried out by Rumiantcev et al. or Minguez-Gabina et al., could be a useful complement to animal or cell experiments in order to enable efficient basic research [139], [140].

## 8 Bone marrow dosimetry

Reliable and efficient bone marrow dosimetry is still an open research field and one of the major challenges in dosimetry. Blood-based dosimetry, where the blood TAC in combination with the red-bone-marrow-to-blood ratio (RMBLR) is used as a surrogate for the TAC in the red bone marrow, is still a commonly applied method [141], [142]. However, this approach assumes that no specific binding to bone marrow cells is present, while image-based bone marrow dosimetry is recommended in case of specific uptake. Specific binding to hematopoietic stem and progenitor cells has been reported for SSTR-targeting radioligands [143], [144], whereupon these cells constitute less than 1 % of cells. However, a major challenge with image-based approaches is that the red bone marrow, and individual cell types in particular, are distributed heterogeneously on the micro-scale [145]. This means that they cannot be imaged directly due to the limited spatial resolution of clinical molecular and anatomical imaging. Thus, in image-based bone marrow dosimetry, VOIs over an entire macroscopic bone region are usually used as a surrogate for dosimetry [142]. Further, the patient-specific distribution of the red bone marrow and the actual mass of red bone marrow in a specific bone region is usually not known, although it is known to be highly dependent on patient-specific factors such as age or presence of bone lesions [70], [146]. Recent efforts aimed at quantifying the patient-specific distribution of red bone marrow by means of magnetic resonance imaging and dual-energy quantitative CT [147], [148], [149]. Other studies investigated molecular imaging techniques to localize the red bone marrow, such as [^99m^Tc]Tc-anti-granulocyte antibody SPECT/CT or [^99m^Tc]Tc-sulfur colloid SPECT/CT, although quantification of the actual target mass for absorbed dose calculation is less straight forward with molecular imaging [70], [150].

The fact that the red bone marrow is distributed throughout the whole-body necessitates improvements towards whole-body SPECT imaging, but also efficient and reliable whole-body dosimetry workflows, including improvements in automatic segmentation and robust co-registration. The situation is further complicated in the presence of bone lesions, which can cause an overestimation of the absorbed dose to the red bone marrow due to partial volume effects. Still, in recent years, significant progress has already been achieved in establishing image-based and clinically feasible approaches for red bone marrow dosimetry for ^177^Lu-PRRT and [^177^Lu]Lu-PSMA therapy [15], [16], [17], [21], [70], [150], [151], [152], including the use of AI-assisted segmentation and micro-models coupled to Monte Carlo simulation workflows [21], [70], [150].

## 9 Approaches for dosimetry-based RLT

PSMA-targeting RLTs and PRRT are typically administered using fixed activities (normally 7.4 GBq) and standardized dosing schedules, i.e. repeated activity administrations in intervals of six weeks for PSMA-targeted RLT up to six treatment cycles, and intervals of eight weeks for PRRT up to four cycles [153], [154]. However, as patients show significant differences in radiopharmaceutical uptake and clearance in both, tumours as well as organs-at-risk, fixed activity schedules may therefore undertreat some patients, while exposing others to avoidable toxicity. In a prospective study by Garske-Román et al., 200 patients underwent multiple cycles of [^177^Lu]Lu-DOTATATE therapy until the absorbed dose to the kidneys exceeded 23 Gy or the absorbed dose to the bone marrow exceeded 2 Gy, or until therapy was discontinued for reasons unrelated to dosimetry [155]. Remarkably, even with a conservative kidney tolerance of 23 Gy, 68.5% of patients could receive more than the standardized four treatment cycles. Overall, an absorbed dose of 23 Gy to the kidneys was reached in three to nine treatment cycles, each with an administration of 7.4 GBq. This highlights the potential to increase the efficiency of RLT using already existing methods and knowledge. At the same time, the growing evidence for ADERs further support the usefulness of dosimetry-guided RLT [10], [11], [12].

**Fig. 1:**
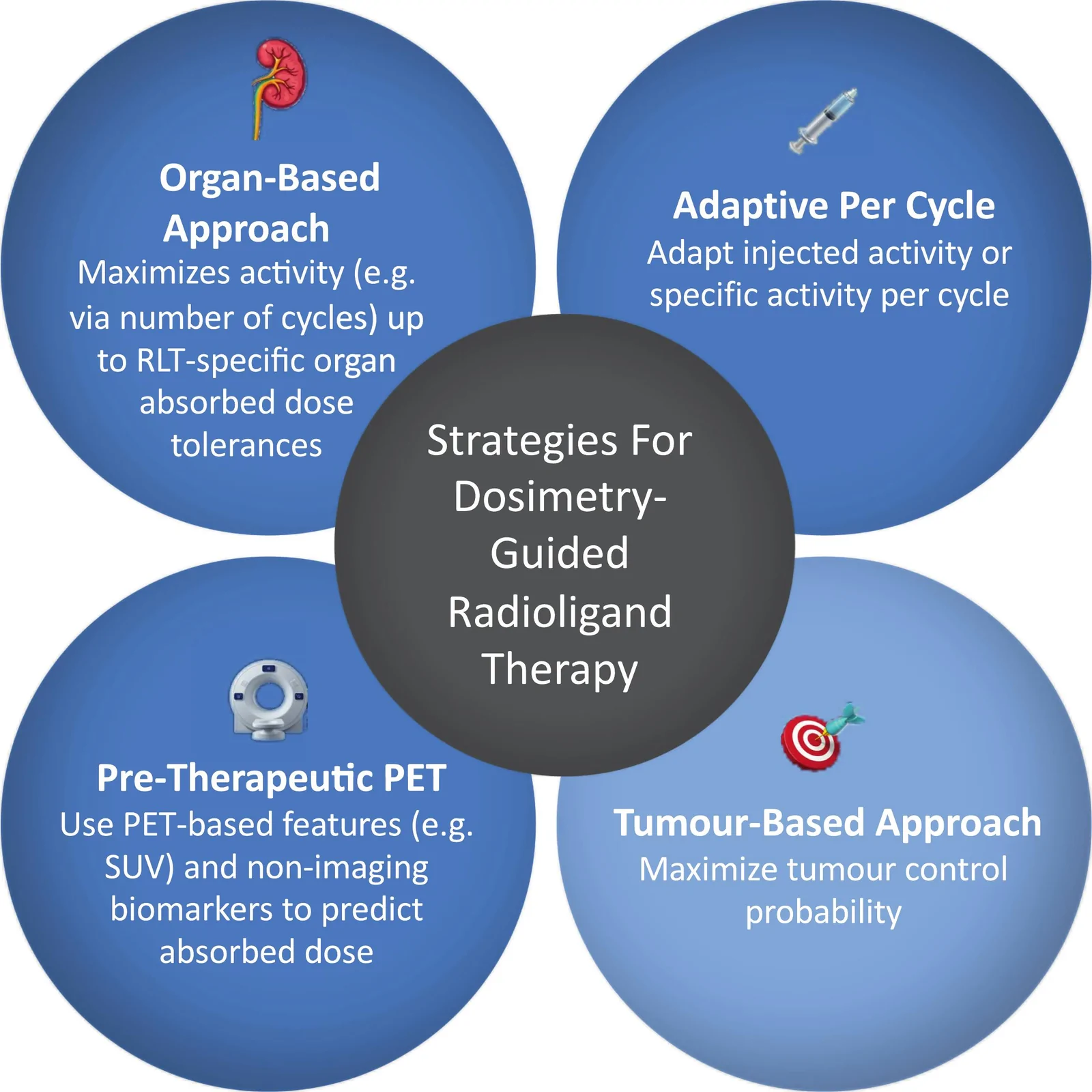
Overview of approaches for dosimetry-guided radioligand therapy

Different approaches for personalized and dosimetry-guided RLT can be considered (Fig. 1). A direct strategy involves personalizing treatment by adapting the number of therapy cycles according to the - ideally RLT-specific - absorbed dose thresholds for organs-at-risk. This approach automatically maximizes tumour absorbed doses according to organ tolerances. This organ-centered approach has already been applied for personalizing PRRT in NET patients, such as the aforementioned study by Garske-Román et al. [155]. In the ILUMINAT trial, 96 patients were treated with [^177^Lu]Lu-DOTATATE until a cumulative absorbed dose of 27 ± 2 Gy was reached in the kidneys [156]. A subgroup of patients received further treatment up to a BED of 40 ± 2 Gy in the kidneys, under the supervision of bone marrow dosimetry. The absorbed dose to the kidneys reached was significantly associated with both progression-free survival and overall survival. The clinical feasibility of an organ-based approach for dosimetry-guided RLT is high, given that STP dosimetry methods have shown good performance, particularly with regard to organ dosimetry. In order to exploit the full potential of organ-based, dosimetry-guided treatment planning, current organ tolerances must be revised specifically for RLT, taking into account both acute and long-term toxicities. This requires the collection of appropriate dosimetric and clinical observational data. Prospective, multi-centre trials with harmonised dosimetry should be established at least in theranostic centres that are already familiar with routine dosimetry.

An alternative approach would be to personalize the injected activity for each cycle rather than using a fixed dosage per cycle. As discussed in the sections above, this approach would also consider changing tumour-to-organ absorbed dose ratios over cycles, as reported in several studies [12], [13], [14]. Further, this approach could specifically adapt to individual organ function and tumour burden, both varying between patients and cycles. For example, Del Prete et al. treated 52 patients with NET using personalized injected activities, with a median activity of 8.8 GBq and a range of 0.7 to 32.4 GBq per cycle [157]. The injected activity was personalized in the following way: in the first cycle, it was determined by the glomerular filtration rate and body surface area, while in subsequent cycles it was adjusted according to the previously observed renal absorbed dose, until the cumulative renal dose reached the limit of 23 Gy. Overall, the tolerability of personalised injected activities observed up to two months after therapy was comparable to that of standard protocols, while higher maximum cumulative tumour absorbed doses were achieved. Future work may also explore adjusting the specific activity to optimize tumour‑to‑organ absorbed dose ratios, as suggested by simulation studies for PSMA ligands [158]. Future clinical trials on the clinical evidence of dosimetry-guided treatment strategies should also consider extended dosimetry concepts such as the BED, that may better reflect the biological effect of altering the activity fractionation schedule compared to the absorbed dose alone. As discussed already, PET-based approaches for predictive dosimetry may assist in selecting the personalised activities already prior to the first treatment cycle.

A key challenge in dosimetry-guided RLT is the large inter-patient variability in treatment response, despite similar tumour absorbed doses, which currently complicates a purely tumour-oriented dosing strategy. This suggests that absorbed-dose-effect-relationships have to be stratified according to, e.g. factors such as DNA repair capacity including the genetic predisposition of the patient, PSMA receptor densities and recycling rates, tissue oxygenation status, ligand internalization and recycling rates, or tumour size and molecular heterogeneity. In-silico studies (e.g. using physiologically-based pharmacokinetic modelling) can assist in better understanding these factors, and guide establishment of respective clinical trials. For example, Begum et al. investigated how ligand amount, binding affinity and internalization rates of PSMA ligands affect both the imaging signal and the therapeutic absorbed dose [159].

## 10 Conclusion

Over the years, considerable advances have been achieved in patient-specific dosimetry for radioligand therapy, providing growing evidence for absorbed-dose-effect-relationships and dosimetry-based activity prescription. However, its clinical implementation remains limited, primarily due to logistical challenges, a lack of standardisation and inadequate reimbursement. Nevertheless, recent developments in quantitative imaging, more robust personalized dosimetry based on lower amount of imaging time points or absorbed dose predictions based on pre-therapeutic PET scans are paving the way towards the broader accessibility of reasonably accurate, personalised dosimetry and its efficient integration into treatment planning. In parallel, novel radionuclides and ligands, as well as microdosimetry and radiobiology studies, are broadening the therapeutic landscape and role of RLT within cancer treatment. Nevertheless, prospective, multi-centre trials are urgently needed to define reliable organ tolerances, expand knowledge about tumour response and further strengthen the clinical evidence for the usefulness of personalized dosimetry in the advancement of RLT toward precision oncology with fully individualized dosing regimens.

## CRediT authorship contribution statement

**Sandra Resch:** Writing – review & editing, Writing – original draft, Visualization, Supervision, Resources, Project administration, Methodology, Investigation, Formal analysis, Data curation, Conceptualization. **Astrid Delker:** Writing – review & editing, Writing – original draft, Visualization, Supervision, Resources, Project administration, Methodology, Investigation, Formal analysis, Data curation, Conceptualization.

## Declaration of competing interest

The authors declare that they have no known competing financial interests or personal relationships that could have appeared to influence the work reported in this paper.
